# Comparative pharmacokinetic study of two mycophenolate mofetil formulations in stable kidney transplant recipients

**DOI:** 10.1111/j.1432-2277.2012.01475.x

**Published:** 2012-04-16

**Authors:** Gere Sunder-Plassmann, Petra Reinke, Thomas Rath, Andrzej Wiecek, Michal Nowicki, Richard Moore, Jens Lutz, Martina Gaggl, Marek Ferkl

**Affiliations:** 1 Division of Nephrology and Dialysis, Department of Medicine III, Medical University ViennaAustria; 2 Department of Nephrology and Intensive Care Medicine, University Hospital CharitéBerlin, Germany; 3 Department of Nephrology and TransplantationWestpfalz-Klinikum, Kaiserslautern, Germany; 4 Department of Nephrology, Endocrinology and Metabolic Diseases, Medical University of Silesia in KatowicePoland; 5 Department of Nephrology, Hypertension, Transplantation, University of ŁódźŁódź, Poland; 6 Nephrology & Transplant, University Hospital WalesCardiff, United Kingdom; 7 Division of Nephrology, Department of Medicine, University Hospital Rechts der IsarMünchen, Germany; 8 Teva Pharmaceuticals EuropeHarlow, United Kingdom

**Keywords:** generic immunosuppressant, kidney transplantation, mycophenolate mofetil, mycophenolic acid, pharmacokinetics

## Abstract

We compared steady-state pharmacokinetics of mycophenolate mofetil (MMF) – Myfenax® (Teva) and CellCept® (Roche) – in stable kidney transplant recipients (KTRs). This was an international, multi-centre, randomized, open-label, two-treatment, two-sequence crossover study with a 3-month follow-up. We included KTRs at least 12 months post-transplantation with stable renal graft function for at least 3 months. The maintenance treatment consisted of MMF in combination with tacrolimus with or without steroids. At the end of the two treatment periods, 6-h or 12-h PK studies of mycophenolic acid (MPA) were performed. A total of 43 patients (mean age: 50.7 ± 13.5 years; 19 females, 24 males) were randomized. Estimates of test to reference ratios (90% CIs) were 0.959 (0.899; 1.023) h*μg/ml for AUC_(0–tau)_ and 0.873 (0.787; 0.968) μg/ml for *C*_max_. Estimates for AUC_(0–6h)_ were 0.923 (0.865; 0.984) h*μg/ml and 0.985 (0.877; 1.106) μg/ml for *C*_min_. Thus, AUC_(0–tau)_, AUC_(0–6h)_, and *C*_min_ of MPA were within the predefined margins. *C*_max_ was somewhat outside of these margins in this set of patients. The numbers and types of adverse events were not different between the two treatments. The steady-state pharmacokinetics of MPA as well as adverse events are comparable for Myfenax® and CellCept® in tacrolimus-treated stable KTRs. (EudraCT-No.: 2009-010562-31; ClinicalTrials.Gov number: NCT00991510)

## Introduction

Mycophenolate mofetil (MMF) and mycophenolate sodium are licensed for the prevention of solid-organ rejection. The active metabolite of both drugs, mycophenolic acid (MPA), is an inhibitor of inosine 5′-monophosphate dehydrogenase (IMPDH), the rate-limiting enzyme of the *de novo* synthesis of guanine nucleotides in lymphocytes [1,2].

A new generic formulation of MMF, Myfenax® had been developed by Teva Pharmaceuticals (Israel) and was approved for use throughout the European Union on the basis of bioequivalence trials in healthy volunteers [3]. However, pharmacokinetic (PK) data of Myfenax® in stable long-term kidney allograft recipients are lacking. To fill this void, we performed an international, multi-centre crossover study comparing steady-state PKs and adverse events of CellCept® (Roche) and Myfenax® in kidney transplant recipients. The use of MMF together with tacrolimus has been clinical practice in transplant care for several years [4,5], and has also the most effective impact on graft protective Th2 responses *in vitro* [6]. Currently, this combination is not an approved indication in the European Union (EU) for both formulations, Myfenax® and CellCept®. However, as the majority of newly transplanted subjects in the EU are given this immunosuppressive regimen, we included tacrolimus-treated patients in this study, representing the standard use of the product.

## Methods

### Study design

**Figure 1:**
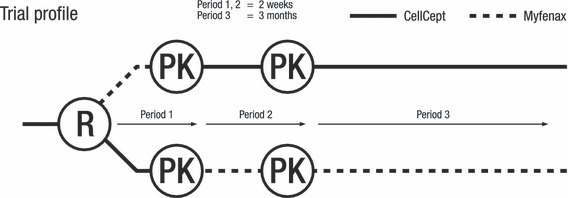
Trial profile.

This was an international, multi-centre, randomized, open-label, two-treatment, two-sequence crossover study with a 3-month follow-up period (Fig. 1). In period I, the subjects received either the test product or the reference product on days 1–14. In period II, the subjects crossed-over to receive the respective other product on days 15–28. In period III, the subjects continued to receive either the test or the reference product until the end of the study (day 112).

On study days 1, 14 (+1 day), 28 (+2 days), 70 (±3 days) and 112 (±3 days), the subjects visited the study centre. Blood samples were taken for determination of MPA trough levels on days 1, 70, and 112. On day 14 and day 28 (under steady-state conditions, i.e., at the end of each cross-over period I + II) blood samples were taken for a full PK profile with samples at time zero (immediately before drug administration, ‘pre-administration’), 30 min, 1 h, 1.5, 2, 3, 4, 5, 6, 8, 10, and 12 h after drug administration. Alternatively, subjects could undergo a shortened 6-h PK profile. For calculation of the full PK profile from the 6-h data, a previously published algorithm [6] was used and validated for our population (data not shown).

All local institutional review boards approved the study and all study participants provided written informed consent.

### Patients

Female and male kidney transplant recipients at least 12 months post-transplantation aged ≥18 years with stable renal graft function (serum creatinine <2.3 mg/dl/<204 μmol/l) for at least 3 months and with no increase in serum creatinine from baseline of more than 0.3 mg/dl for at least 1 month prior to the start of the study were included in the study.

The immunosuppressive maintenance treatment included MMF (CellCept®) in combination with tacrolimus (Prograf®, Astellas), with or without corticosteroids. The dose of MMF was ≥500 mg twice daily with no changes in the immunosuppressive regimen for at least 6 weeks prior to the start of the study.

Female subjects had to be either postmenopausal for ≥1 year, or surgically sterilized, or, if women of childbearing potential, a negative pregnancy test was required immediately prior to study entry and such subjects had to continue to use effective contraception.

### Investigational drugs

The test product was Myfenax® (500 mg tablets and 250 mg capsules) and the reference product was CellCept® (500 mg tablets and 250 mg capsules). The subjects administered tablets and capsules of either product at a dose of at least 500 mg twice daily, morning and evening. On the days of blood sampling for MPA trough levels and for PK studies, the patients received a standardized breakfast and lunch. Daily treatment with test or reference drug was administered for 112 days (±3 days). Thereafter all patients continued to take CellCept®.

### Laboratory analyses

Samples for MPA analysis were immediately centrifuged and frozen at the participating centres, stored at less then −20 °C and shipped frozen by batches to the central laboratory. MPA plasma concentrations were measured there using a validated HPLC method with on-line UV detection [7]. This technique is appropriately sensitive and reliable in order to determine drug levels as low as 0.250 μg/ml in plasma. All other laboratory analyses were performed in local clinical chemistry institutes.

### Statistical analysis

Based on previous single-dose studies, the intra-subject coefficients of variation were 14% and 50% for MPA-AUC and MPA-*C*_max,_ respectively. Based on the literature, similar intra-subject coefficients of variation were observed in steady-state patients. Thus, with these expected coefficients of variation and an expected ratio of *C*_max_ within 0.95 and 1.05, the study should have a power of at least 80% to show comparable bioavailability with 80 subjects. A total of 100 subjects were planned to be enrolled, allowing for >10% dropout rate.

For assessment of comparative bioavailability, 90% confidence intervals (CIs) for the formulation ratio in the parameters AUC_0–tau_ and *C*_max_ of MPA were calculated using the ln-transformed data using data from both period I and II. The PK parameters were assessed against the standard bioequivalence margins of 90% CIs within 0.80–1.25 for AUC_(0–tau)_ and *C*_max_.

Pharmacokinetic variables comprised AUC_0–tau_: For subjects with a 0–12 h profile, an area under the plasma concentration–time curve during a dosage interval at steady state (calculated using the trapezoidal rule, from *t* = 0 to *t* = 12 h); for subjects with a 0–6 h profile: AUC_(0–tau)_ was calculated based on AUC_(0–6h)_ using the extrapolation formula, method D, according to Fleming [8]; AUC_(0–6h)_: area under the plasma concentration–time curve during a dosage interval at steady state (calculated using the trapezoidal rule, from *t* = 0 to *t* = 6 h); *C*_max_: directly obtained from measured values; *C*_min_: directly obtained from measured values at the end of the dosage interval at steady state; *T*_max_: directly obtained from measured values; *C*_pd_: directly obtained from measured values; PTF (degree of fluctuation of the concentration levels over one dosing interval): (*C*_max_ − *C*_min_)/(AUC_τ_/τ)*100.

Safety variables were tabulated with descriptive group statistics. Adverse events (AEs) were coded with the Medical Dictionary for Regulatory Activities MedDRA (version 12.1). Safety variables included physical examination, vital signs (heart rate and blood pressure in sitting position), 12-lead electrocardiogram (ECG) at screening and study termination, documentation of AEs and laboratory data (clinical chemistry, haematology, and urinalysis).

## Results

### Patients

A total of 47 subjects were enrolled in the study (enrolment was terminated before inclusion of all planned patients because of low recruitment rates). Four of forty-seven subjects were not randomized, i.e., two subjects withdrew consent, one subject had a protocol violation, and one subject was a screening failure (did not meet eligibility criteria). 43 subjects were randomized, and 41 subjects completed the study (there were 2 subjects who discontinued the study because of AEs). The safety analysis set consisted of 43 subjects, and the PK analysis set consisted of 41 subjects (no PK data in one subject and protocol violation in the other case). A total of 41 patients completed the 6-h PK profile and 20 patients completed a 12-h PK profile. Important demographic and baseline data, as well as relevant concomitant medication are indicated in Table 1.

**Table 1:** Demography, baseline characteristics, and concomitant medication (safety population, *n* = 43).

Age (years)	50.7 ± 13.5
Sex (female/male)	19 / 24
Body mass index (kg/m^2^)	26.1 ± 3.56
Race (Caucasian/Asian)	42/1
Diabetes mellitus	7
Tacrolimus use	43
Serum creatinine (μmol/l)	116 ± 28
Steroid use	22
Drugs for acid-related disorders	28

Data are given as mean ± SD or as absolute numbers.

### Pharmacokinetic analysis

**Figure 2:**
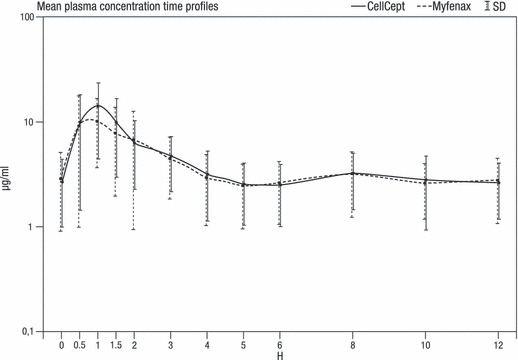
Plasma concentration time profile of MPA.

Mean plasma concentrations of MPA increased from pre-administration to 1 h after administration and decreased thereafter, reaching pre-administration levels at 5 h after administration in both treatment groups (Fig. 2). Descriptive statistics of plasma concentrations of MPA over time are summarized in Table 2.

**Table 2:** Plasma concentration of MPA by treatment and time point (PK population, *n* = 41)

Sampling time	CellCept®	Myfenax®
Pre-administration	2.69 ± 1.7 (0.50–7.26)	3.00 ± 2.09 (0.54–8.72)
0.5 h	9.78 ± 8.35 (1.77–39.64)	9.37 ± 8.38 (0.45–36.35)
1.0 h	13.94 ± 9.52 (2.59–48.90)	10.51 ± 6.52 (0.29–25.29)
1.5 h	9.81 ± 6.83 (0.70–29.89)	7.86 ± 5.89 (0.26–31.73)
2.0 h	6.26 ± 3.99 (1.42–18.51)	6.78 ± 5.84 (0.25–24.43)
3.0 h	4.69 ± 2.52 (0.99–11.89)	4.51 ± 2.68 (0.32–11.91)
4.0 h	3.18 ± 2.05 (0.73–11.89)	2.95 ± 1.92 (0.70–10.35)
5.0 h	2.54 ± 1.50 (0.34–7.67)	2.46 ± 1.50 (0.65–6.79)
6.0 h	2.48 ± 1.47 (0.46–6.31)	2.62 ± 1.57 (0.48–6.81)
8.0 h	3.26 ± 1.80 (0.50–8.64)	3.20 ± 1.97 (0.61–7.19)
10.0 h	2.84 ± 1.89 (0.44–7.38)	2.60 ± 1.41 (0.47–7.42)
12.0 h	2.62 ± 1.44 (0.61–5.92)	2.79 ± 1.71 (0.70–7.42)

Data in μg/ml given as mean ± SD (minimum–maximum).

Pharmacokinetic parameters were analyzed for two populations. The primary analysis included all subjects of the PK population (*n* = 41). The sensitivity analysis excluded three subjects with incomplete or questionable PK profiles (2 on Myfenax® and 1 on CellCept®). Descriptive statistics for both analysis sets are provided in Tables 3 and 4.

**Table 3:** Summary of PK data (PK population, *n* = 41).

Parameter	CellCept®	Myfenax®
AUC _(0–6 h)_	33.52 ± 15.13 (10.20–67.34)	31.10 ± 15.42 (11.21–84.95)
AUC _(0–tau)_	49.85 ± 20.83 (13.44–93.46)	48.26 ± 21.22 (15.68–111.89)
*C*_max_	16.19 ± 9.95 (3.49–48.90)	14.31 ± 8.34 (3.72–36.35)
*C*_min_	1.58 ± 0.78 (0.34–3.65)	1.57 ± 0.74 (0.25–3.18)
*C*_predose_	2.69 ± 1.70 (0.50–7.26)	3.00 ± 2.09 (0.54–8.72)
PTF	351.1 ± 161.2 (67.3–809.5)	323.7 ± 156.0 (84.1–707.8)
*T*_max_	1.12 ± 0.75 (0.00–3.78)	1.34 ± 1.14 (0.17–5.92)

Data are indicated as mean ± SD (minimum–maximum). AUCs are given as h^*^μg/ml, Cs are given as μg/ml, *T*_max_ is given in hours.

**Table 4:** Summary of PK data (sensitivity analysis, *n* = 38).

Parameter	CellCept®	Myfenax®
AUC_(0–6 h)_	33.75 ± 15.26 (10.20–67.34)	30.77 ± 15.70 (11.21–84.95)
AUC_(0–tau)_	50.07 ± 21.06 (13.44–93.46)	47.31 ± 21.29 (15.68–111.9)
*C*_max_	16.58 ± 10.18 (3.49–48.90)	14.38 ± 8.50 (3.72–36.35)
*C*_min_	1.57 ± 0.80 (0.34–3.65)	1.56 ± 0.72 (0.47–3.18)
*C*_predose_	2.72 ± 1.75 (0.50–7.26)	2.84 ± 1.86 (0.60–8.72)
PTF	359.6 ± 164.4 (67.3–809.5)	326.2 ± 159.8 (84.1–707.8)
*T*_max_	1.12 ± 0.75 (0.00–3.78)	1.28 ± 0.89 (0.45–3.95)

Data are indicated as mean ± SD (minimum-maximum). AUCs are given as h^*^μg/ml, Cs are given as μg/ml, *T*_max_ is given in hours.

The PK comparison shows estimates (90% CIs) of 0.959 (0.899; 1.023) h*μg/ml for AUC_(0–tau)_, and of 0.873 (0.787; 0.968) μg/ml for *C*_max_. Estimates (90% CIs) for AUC_(0–6h)_ were 0.923 (0.865; 0.984) h*μg/ml, and 0.985 (0.877; 1.106) μg/ml for *C*_min_. Comparable bioavailability was accepted if the calculated 90% CIs were within 0.80–1.25. Thus, AUC_(0–tau)_, AUC_(0–6h)_, and *C*_min_, were within the predefined margins, but not *C*_max._ The results of the PK comparison are summarized in Table 5. Results of the sensitivity analysis were very similar.

**Table 5:** Pharmacokinetic comparison of Myfenax® and CellCept® (anova of non-zero, logarithmized parameters).

Parameter	Alpha estimate	(90% CI)
Primary analysis (PK population)		
AUC _(0–6 h)_	0.1	0.923 (0.865; 0.984)
AUC _(0–tau)_	0.1	0.959 (0.899; 1.023)
*C*_max_	0.1	0.873 (0.787; 0.968)
*C*_min_	0.1	0.985 (0.877; 1.106)
Sensitivity analysis		
AUC _(0–6 h)_	0.1	0.908 (0.852; 0.968)
AUC _(0–tau)_	0.1	0.942 (0.884; 1.004)
*C*_max_	0.1	0.859 (0.772; 0.956)
*C*_min_	0.1	1.017 (0.921; 1,123)

AUCs are given as h^*^μg/ml, Cs are given as μg/ml.

### Safety analysis

Overall, 26 patients experienced 69 adverse events (AEs). Nine subjects experienced 11 AEs considered related to the study drug. In the CellCept® group, the study drug-related events were peripheral edema, herpes simplex, and bowenoid papulosis. In the Myfenax® group, the related events were diarrhea, herpes zoster, lower respiratory tract infection, urinary tract infection, headache (2 subjects), tremor, and hypertension. These events were not unexpected and a causal relationship to the study drugs is not certain. In three patients AE resulted in discontinuation of the study drug. All three listed serious adverse events (SAEs) were recorded in one individual who was hospitalized twice because of atrial fibrillation and heart failure as a consequence of another episode of atrial fibrillation. Each event happened in a different treatment period, thus concerning both the test and the reference formulation. There were no AEs of severe intensity and no deaths. In general, the AE profile was similar between the CellCept® and the Myfenax® treatment groups. Most common AEs were gastrointestinal complaints (20 events in 11 patients) and infections (11 events in 10 patients). There was no relevant change or abnormal values in any of the laboratory parameters obtained during the study in both treatment groups. A comparison of AEs in both treatment groups is provided in Table 6.

**Table 6:** Summary of adverse events (safety population, *n* = 43).

	Number of events (number of patients)			
	CellCept®	Myfenax®	Overall
All recorded AEs	32 (15)	37 (17)	69 (26)
AEs considered related	3 (3)	8 (7)	11 (9)
Severe AEs	0	0	0
AEs leading to discontinuation	3 (2)	2 (1)	5 (3)
Serious AEs	2 (1)	1 (1)	3 (1)
AEs leading to death	0	0	0

AE, adverse event.

## Discussion

Mycophenolic acid represents a cornerstone of successful immunosuppression after kidney transplantation, in the short-term [5], as well as in long-term treatment [9]. With the present study, we provide evidence that the steady state PKs of MPA, after administration of Myfenax®, a generic form of MMF, or CellCept®, the originator drug, are comparable in stable kidney allograft recipients.

Immunosuppressive therapy of transplant recipients results in high costs for the individual, health care systems and society. However, generic drugs have the potential of providing equivalent therapeutic efficacy at a lower cost [10]. Therefore, the American Society of Transplantation has organized a consensus meeting where participants strongly supported the availability of less expensive medications and the introduction of generic alternatives [11]. Besides the decrease of financial burdens, another reason for this consensus was the assumption that medication costs can contribute to noncompliance with prescribed immunosuppressive regimens. Consequently, the American Society of Transplantation argued for the use of generic immunosuppressants in order to decrease financial burdens, requiring that the equivalent therapeutic efficacy of marketed generics is approved by regulatory authorities [11], as did the European Society for Organ Transplantation most recently [12].

Nevertheless, there may be some need for clinical studies that assess generic immunosuppressive drugs because many transplant physicians feel more comfortable in using such medications with a background of clinical data obtained from transplant recipients. For cyclosporine A [13,14] and tacrolimus [15], such trials have recently been published. In the case of MMF, to the best of our knowledge, no such studies are available.

This postapproval study was performed to compare steady state PKs of two MMF containing products in subjects with stable renal transplants under real-life clinical conditions. Although only 43 renal transplant subjects (instead of 100 patients initially planned) had been enrolled in this study to compare Myfenax® and CellCept®, the pharmacokinetic results for both products in terms of AUC_(0–tau)_, AUC_(0–6h)_, and *C*_min_ were within the preset margins (CI 80–125%). It is worth to mention that we validated the algorithm introduced by Fleming *et al.* [8] for estimating 12-h PK profiles from 6-h data in the subset of our patients that had full PK profiles for Myfenax® and CellCept® (data not shown). The *C*_max_ results showed a higher variability, confirming those from previous studies, with the lower limit of the 90% confidence interval below the predefined margin in this set of patients. In this context, the PKs of MPA have shown high between-subject and within-subject variability with a more than 10-fold range of MPA dose normalized AUCs in patients after solid organ transplantation [16–18], as well as in healthy individuals [19]. This between-subject variability has been attributed to differences in albumin concentration, bilirubin and haemoglobin concentrations, renal and liver function, co-administration of other drugs (e. g. cyclosporine A, steroids, proton pump inhibitors), co-morbidities, body weight, age, and gender [16]. Genetic polymorphisms of the drug metabolizing enzymes [20] as well as of the drug target, inosine 5′-monophosphate dehydrogenase [21] have also substantial effects on the PKs and pharmacodynamics of MPA.

Within-subject variability of MPA exposure is less well described, and somewhat smaller than between-subject variability, as has also been observed in one of our previous studies [22]. Mean intra-individual coefficients of variation of *C*_predose_, AUC_0–12 h_, and *C*_max_, however, exceeded 30%, being highest for the latter one [18,19,23,24] in both healthy individuals and kidney transplant recipients.

With regard to safety, there were no AEs of severe intensity and no deaths. There was one subject with SAEs, which were unrelated to the study drug. In general, the AE profile was similar between the CellCept® and Myfenax® treatment groups. Pre/post comparisons showed no relevant changes or abnormalities regarding safety laboratory, vital signs, physical examination or ECG results (data not shown) during the crossover treatment period and the follow-up phase. Both formulations were well tolerated and gastrointestinal symptoms were the most frequently reported side effects [25].

A limitation to our study stands on the fact that we terminated the trial before all planned patients could be included. However, even with the lower than calculated patient numbers, we demonstrated AUCs within the pre-specified limits and Cmax only marginally out of these limits.

In summary, the present study shows that the steady-state pharmacokinetics of mycophenolic acid as well as the adverse events profiles are comparable for Myfenax® and CellCept® in tacrolimus-treated stable kidney transplant recipients.

## Authorship

GS-P and MF: designed the study. GS-P, PR, TR, AW, MN, JL and MG: performed the study. GS-P, PR, TR, AW, MN, JL, MG and MF: wrote the manuscript.

## Funding source

Teva Europe.
